# Ferroptosis contributes to isoflurane-induced neurotoxicity and learning and memory impairment

**DOI:** 10.1038/s41420-021-00454-8

**Published:** 2021-04-07

**Authors:** Pengfei Liu, Jing Yuan, Yetong Feng, Xin Chen, Guangsuo Wang, Lei Zhao

**Affiliations:** 1 Ambulatory Surgical Center, The Second Clinical Medical College, Jinan University (Shenzhen People’s Hospital), 518020 Shenzhen, China; 2 The First Affiliated Hospital, Jinan University, 510632 Guangzhou, China; 3 School of Pharmaceutical Sciences, Jilin University, 130012 Changchun, China; 4 Department of Laboratory Medicine, The Second Clinical Medical College, Jinan University (Shenzhen People’s Hospital), 518020 Shenzhen, China; 5 Department of Thoracic Surgery, The Second Clinical Medical College, Jinan University (Shenzhen People’s Hospital), 518020 Shenzhen, China

**Keywords:** Hippocampus, Neonatal brain damage

## Abstract

Ferroptosis is a novel type of programmed cell death, which is different from apoptosis and autophagic cell death. Recently, ferroptosis has been indicated to contribute to the in vitro neurotoxicity induced by isoflurane, which is one of the most common anesthetics in clinic. However, the in vivo position of ferroptosis in isoflurane-induced neurotoxicity as well as learning and memory impairment remains unclear. In this study, we mainly explored the relationship between ferroptosis and isoflurane-induced learning and memory, as well as the therapeutic methods in mouse model. Our results indicated that isoflurane induced the ferroptosis in a dose-dependent and time-dependent manner in hippocampus, the organ related with learning and memory ability. In addition, the activity of cytochrome c oxidase/Complex IV in mitochondrial electron transport chain (ETC) was increased by isoflurane, which might further contributed to cysteine deprivation-induced ferroptosis caused by isoflurane exposure. More importantly, isoflurane-induced ferroptosis could be rescued by both ferroptosis inhibitor (ferrostatin-1) and mitochondria activator (dimethyl fumarate), which also showed effective therapeutic action against isoflurane-induced learning and memory impairment. Taken together, our data indicate the close association among ferroptosis, mitochondria and isoflurane, and provide a novel insight into the therapy mode against isoflurane-induced learning and memory impairment.

## Introduction

Isoflurane is considered as one of the most commonly used volatile anesthetics all over the world, because of its high efficiency and controllability^1^. In recent years, more and more evidences indicated that the exposure to isoflurane, or even other anaesthetics (e.g., sevoflurane, midazolam, and thiopental), can lead to neurotoxicity to the developing brain in both experimental animal models and clinical cases^2–5^. Moreover, the anesthetic exposure is also correlated with the persistent learning and memory deficits^6–8^. So far the neurotoxicity induced by isoflurane is only limited to the development period, and no obvious neurotoxic effects can be found in adult brain^5,9^. Therefore, it’s necessary to develop clinically protective strategy against isoflurane-induced neurotoxicity as well as learning and memory impairment in pediatric surgery.

Various mechanisms have been proposed for isoflurane-induced neurotoxicity in recent years. For example, some studies link isoflurane-induced neurotoxicity with RhoA-mediated cytoskeletal depolymerization. However, the inhibition of the process is still hard to rescue the neural impairment^10,11^. In addition, the apoptosis of neural cells has emerged as a key factor in isoflurane-induced neurotoxicity, and some studies indicate that isoflurane-induced Caspase 3 activation in neural cells contributes to the impairment of learning and memory ability, and the suppression of apoptosis enhances the learning and memory ability significantly^12–14^. Thus, the anti-apoptotic agents may holds great potential against isoflurane-induced neurotoxicity in clinic in the future.

In 2019, some scientists found that ferroptosis also contributed to isoflurane-induced neurotoxicity besides apoptosis^15,16^. Different from apoptosis or autophagic cell death, ferroptosis is a novel form of programmed cell death, and characterized by iron-dependent accumulation of lipid peroxidation and reactive oxygen species (ROS)^17^. Glutathione (GSH) is an important regulator in ferroptosis, and the inhibition of cystine-glutamate antiporter (system Xc^−^) can lead to the depletion of cellular cysteine and GSH, which disrupts cellular redox homeostasis and results in ferroptosis eventually. On the other side, glutathione peroxidase 4 (GPX4) and ferroptosis suppressor protein 1 (FSP1) are also considered as important inhibitors for ferroptosis, and the inhibition of GPX4 or FSP1 influences the clearance of lipid ROS and induces ferroptosis without affecting the regular levels of cysteine or GSH^18–21^. Therefore, the regulations of ferroptosis induced by different reasons could be varied absolutely. For example, mitochondria holds a close correlation with ferroptosis, and both mitochondrial damage and mitochondrial ROS production are related with the process of ferroptosis. However, mitochondrial electron transport chain (ETC) activity and tricarboxylic acid (TCA) cycle are only essential for lipid ROS production in cysteine deprivation-induced ferroptosis, but not in GPX4 inhibition-induced ferroptosis, and the inhibition of mitochondrial ETC activity or TCA cycle only relieves the ferroptosis caused by cystine starvation^22,23^.

In recent years, ferroptosis has been demonstrated to hold an important position in kinds of diseases, such as cancer^24,25^, kidney injury ^26,27^, lung injury^28,29^ and neurological diseases^30,31^. Moreover, the blockage or activation of ferroptosis has also been considered as a promising therapeutic strategy^32–34^. Even though some evidences have indicated that ferroptosis is involved in the isoflurane-induced neurotoxicity in vitro^15,16^, the in vivo significance of ferroptosis in isoflurane-induced neurotoxicity as well as learning and memory impairment remains unclear. In this study, our results mainly indicated that isoflurane exposure induced ferroptosis in hippocampus, the organ related with learning and memory. In addition, isoflurane also increased the activity of cytochrome c oxidase/Complex IV in mitochondrial ETC, which contributed to cysteine deprivation-induced ferroptosis. More importantly, the pre-treatment with either ferroptosis inhibitor (ferrostatin-1) or mitochondria protective agent (dimethyl fumarate) relived isoflurane-induced ferroptosis as well as learning and memory impairment, providing a novel therapeutic strategy against the neurotoxicity induced by isoflurane.

## Results

### Isoflurane exposure induces ferroptosis in hippocampus

**Fig. 1:**
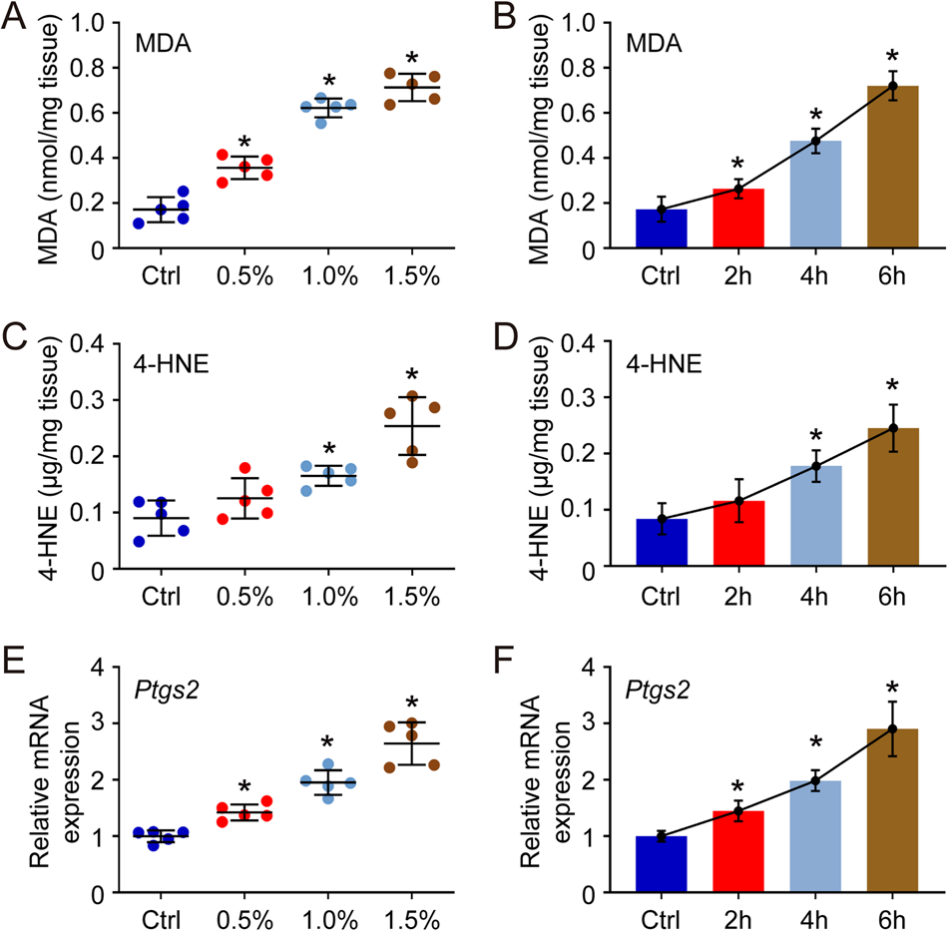
Effect of isoflurane exposure on ferroptosis in hippocampus. In does-dependent assay, the mice received 0.5%, 1.0% and 1.5% isoflurane exposure for 6 h, while in time-dependent assay, the mice received 1.5% isoflurane exposure for 2 h, 4 h and 6 h respectively. Then, all of mice were sacrificed immediately, and the hippocampus was isolated for different measurements. Herein, the level of MDA (**A**, **B**) and 4-HNE (**C**, **D**), as well as the expression of *Ptgs2* (**E**, **F**), were measured to evaluate the ferroptosis in each group. *p value < 0.05 compared with the Ctrl group.

Since hippocampus is considered as the most important organ related with learning and memory ability, the effect of isoflurane (0.5%, 1.0% and 1.5% isoflurane exposure for 6 h, or 1.5% isoflurane exposure for 2 h, 4 h and 6 h respectively) on ferroptosis in mouse hippocampus was mainly analyzed in our study. The level of MDA and 4-HNE were measured first, and the results indicated that isoflurane exposure increased the level of both MDA and 4-HNE in hippocampus in a dose-dependent and time-dependent manner (Fig. 1A–D). In addition, the expression of *Ptgs2* (a marker gene of ferroptosis) was measured using qPCR, and the results were similar to previous MDA and 4-HNE assay. The expression of *Ptgs2* was upregulated by isoflurane exposure obviously (Fig. 1E, F). Totally, those results indicated that isoflurane exposure indeed induced ferroptosis in hippocampus.

### Isoflurane induces ferroptosis via affecting system Xc^−^ in hippocampus

**Fig. 2:**
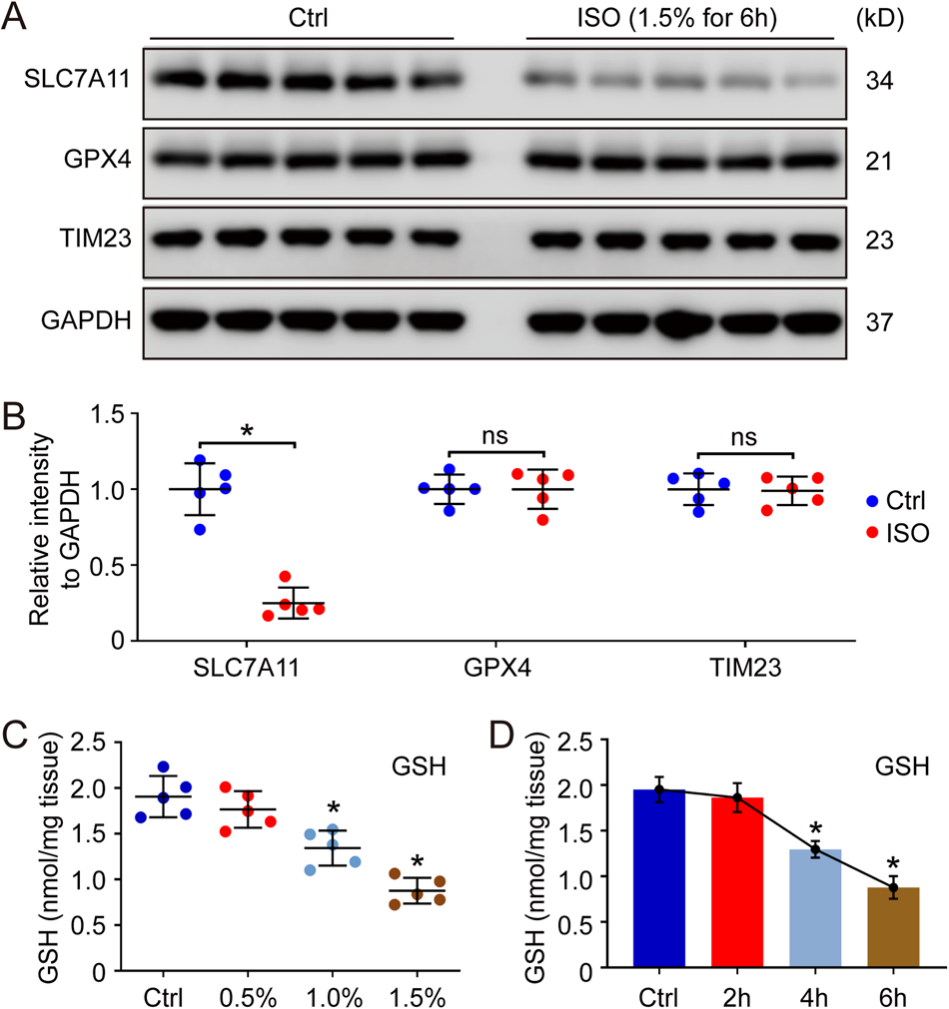
Isoflurane induces ferroptosis via affecting system Xc^−^ in hippocampus. After isoflurane exposure (ISO, 1.5%) for 6 h, the hippocampus was isolated for western blot detection (**A**). The quantification of western blot was showed in (**B**). Moreover, the effect of isoflurane exposure on GSH level in hippocampus was also evaluated in different groups (**C**, **D**). In **B**, *p value < 0.05 compared between two groups. ns: *p* value > 0.05 compared between two groups. In **C**, **D**, *p value < 0.05 compared with the Ctrl group.

Some study indicated that isoflurane could induce ferroptosis in primary neuronal cells in vitro via inhibiting the expression of GPX4^16^. Therefore, the effect of isoflurane exposure on GPX4 was also evaluated in our study. Different from the report, the level of GPX4 didn’t show any change in hippocampus after isoflurane exposure (1.5% isoflurane exposure for 6 h). However, the protein level of SLC7A11, a key factor in system Xc^−^, showed an obvious downregulation after isoflurane exposure (Fig. 2A, B). We also measured the level of GSH, the downstream of system Xc^−^, and the results also suggested that isoflurane decreased GSH level in hippocampus in both dose-dependent and time-dependent manner (Fig. 2C, D), which is consistent with SLC7A11 assay. Therefore, it’s possible that isoflurane exposure induces ferroptosis in hippocampus via affecting system Xc^−^ and cysteine uptake, but not GPX4.

### Isoflurane exposure increases the activity of cytochrome c oxidase/Complex IV in mitochondrial ETC

**Fig. 3:**
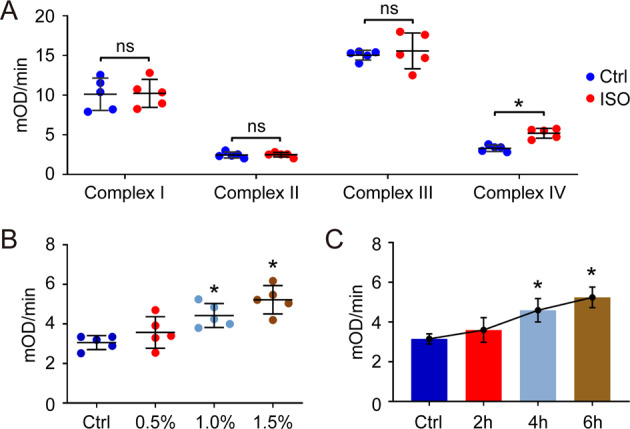
Analysis of the activity of Complex I–IV in mitochondrial ETC. After isoflurane exposure (ISO, 1.5%) for 6 h, the hippocampus was isolated for the analysis (**A**). The effect of isoflurane exposure on the activity of cytochrome c oxidase/Complex IV in mitochondrial ETC was also evaluated in does-dependent assay (**B**) and time-dependent assay (**C**). In **A**, *p value < 0.05 compared between two groups. ns: *p* value > 0.05 compared between two groups. In **B**, **C**, *p value < 0.05 compared with the Ctrl group.

Some reports have indicated that isoflurane leads to mitochondrial dysregulation in the developing brain^35,36^. Moreover, the mitochondrial ETC activity is also essential for lipid ROS production in cysteine deprivation-induced ferroptosis^22^. Thus, the activity of different complexes in mitochondrial ETC were tested in our research. Our results showed that the level of mitochondrial protein, TIM23, was similar between the ctrl group and isoflurane exposure group, indicating that the total mitochondria amount in hippocampus wasn’t influenced by isoflurane (Fig. 2A, B). In addition, the isoflurane exposure didn’t affect the activity of Complex I–III in hippocampus. However, the activity of cytochrome c oxidase/Complex IV in mitochondrial ETC was increased by isoflurane (Fig. 3A). To further confirm this results, we measured the effect of isoflurane exposure (0.5%, 1.0% and 1.5%) on the activity of cytochrome c oxidase/Complex IV in different time points (2 h, 4 h and 6 h). The results also indicated that isoflurane exposure enhanced the activity of cytochrome c oxidase/Complex IV in hippocampus in both dose-dependent and time-dependent manner (Fig. 3B, C), which might contribute to the cysteine deprivation-induced ferroptosis.

### Ferrostatin-1 (Fer-1) and dimethyl fumarate (DMF) rescue isoflurane-induced ferroptosis

**Fig. 4:**
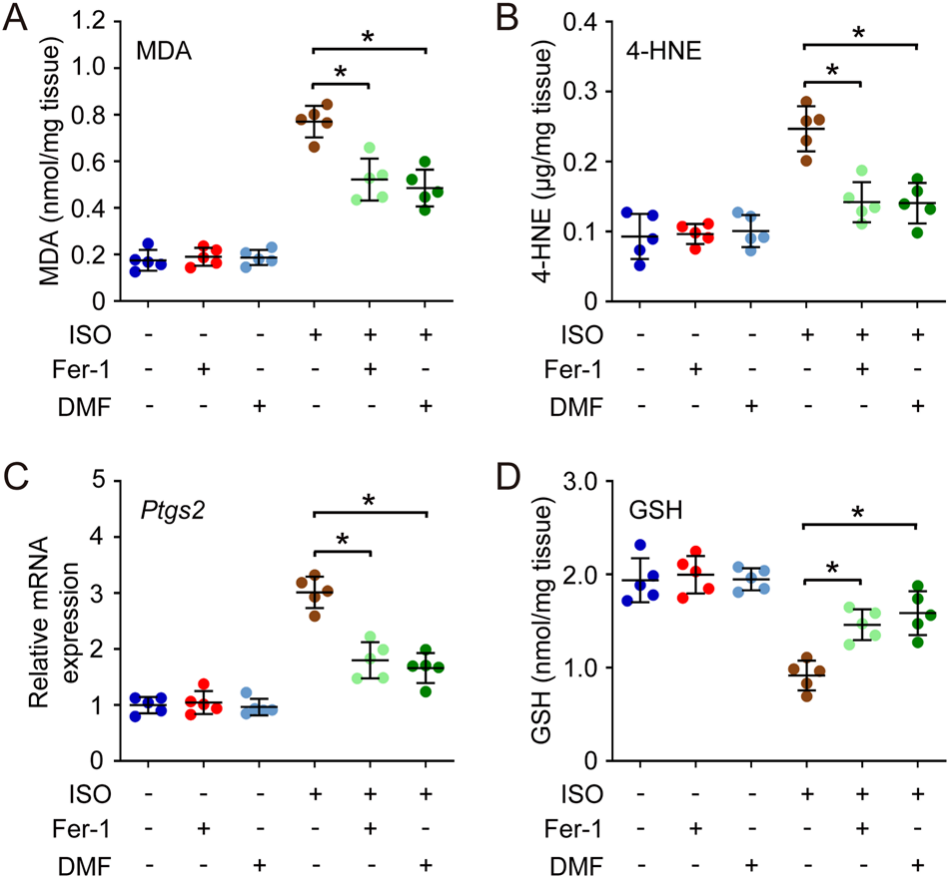
Pre-treatment with Fer-1 and DMF rescue isoflurane-induced ferroptosis. In the pre-treatment, 15 mg/kg DMF was administered (i.p.) 16 h before anesthesia, and 1 pmol of Fer-1 was injected into the striatum immediately before anesthesia. Then, the mice received isoflurane exposure (ISO, 1.5%) for 6 h, and the hippocampus was isolated for the analysis. Herein, the level of MDA (**A**) and 4-HNE (**B**), the expression of *Ptgs2* (**C**), as well as the level of GSH (**D**), were measured to evaluate the ferroptosis in each group. *p value < 0.05 compared between two groups.

To relieve the ferroptosis and mitochondrial dysregulation caused by isoflurane exposure, the mice were pre-treated with Fer-1 (ferroptosis inhibitor) or DMF (mitochondria protective agent), then exposed to isoflurane (1.5%) for 6 h. We found the pre-treatment with either Fer-1 or DMF didn’t affect the basal level of MDA and 4-HNE. However, after isoflurane exposure, the level of MDA and 4-HNE in the pre-treated groups were much lower than untreated group (Fig. 4A, B). Similar results were found with the *Ptgs2* expression (Fig. 4C). In addition, the level of GSH in hippocampus was also improved by Fer-1 or DMF compared with isoflurane exposure group (Fig. 4D).

**Fig. 5:**
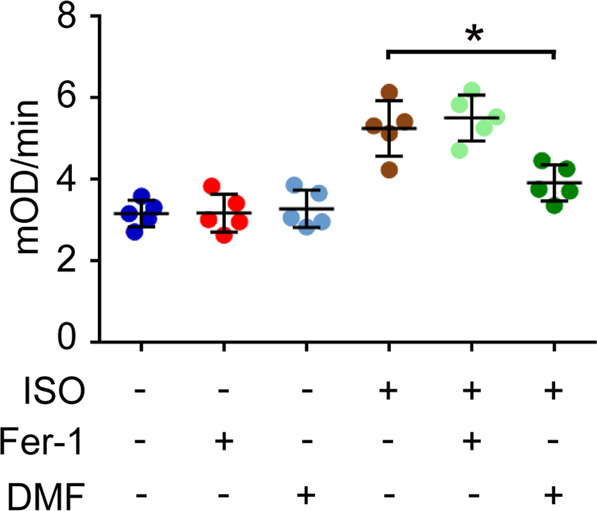
Pre-treatment with DMF relieves the influence of isoflurane on the activity of cytochrome c oxidase/Complex IV in mitochondrial ETC. In the pre-treatment, 15 mg/kg DMF was administered (i.p.) 16 h before anesthesia, and 1 pmol of Fer-1 was injected into the striatum immediately before anesthesia. Then, the mice received isoflurane exposure (ISO, 1.5%) for 6 h, and the hippocampus was isolated for the analysis. Herein, the activity of cytochrome c oxidase/Complex IV in mitochondrial ETC was evaluated in each group. *p value < 0.05 compared between two groups.

The activity of cytochrome c oxidase/Complex IV in mitochondrial ETC was also measured in different groups. Even though Fer-1 rescued the isoflurane-induced ferroptosis, the enhanced activity of Complex IV in mitochondrial ETC wasn’t affected by Fer-1 treatment. However, the treatment with DMF decreased the activity of Complex IV in mitochondrial ETC obviously (Fig. 5). Therefore, DMF might inhibit ferroptosis through regulating mitochondrial function, and the mechanisms for the therapeutic action of Fer-1 and DMF against isoflurane-induced ferroptosis were different from each other.

### Both Fer-1 and DMF relieve isoflurane-induced learning and memory impairment

**Fig. 6:**
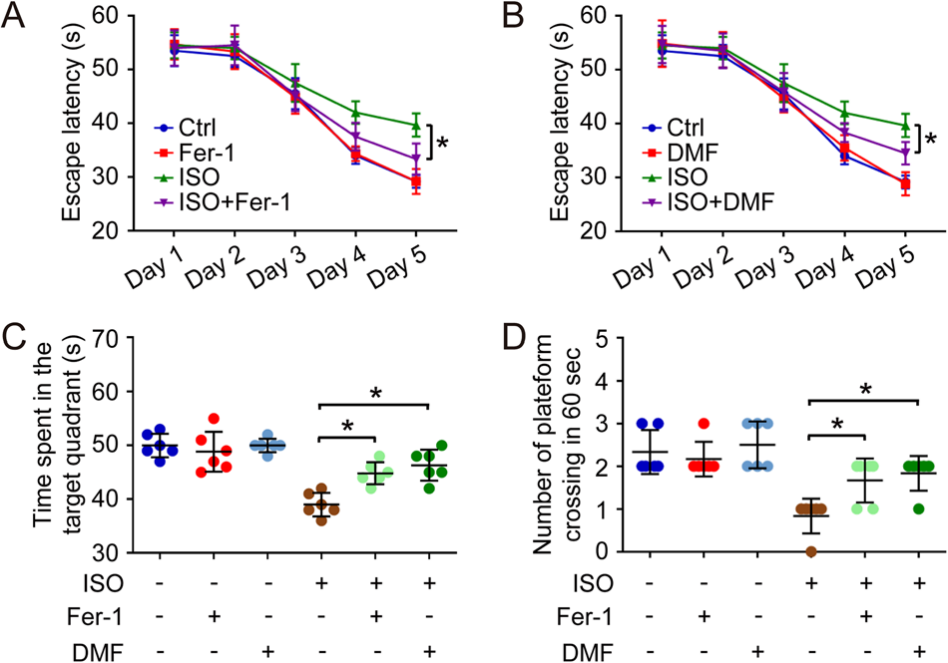
Fer-1 and DMF relieve isoflurane-induced learning and memory impairment. In the pre-treatment, 15 mg/kg DMF was administered (i.p.) 16 h before anesthesia, and 1 pmol of Fer-1 was injected into the striatum immediately before anesthesia. Then, the mice received isoflurane exposure (ISO, 1.5%) for 6 h. 3 weeks later, the learning and memory ability of the mice in different groups were analyzed using Morris water maze test. Herein, the escape latency (**A**), the time spent in the target quadrant (**B**), and the number of plateform crossing in 60 sec (**C**) were calculated in each group. *p value < 0.05 compared between two groups.

Some studies have revealed that the isoflurane-induced neurotoxicity can lead to the impairment of learning and memory, the most important features of cognition^14,34,37^. Therefore, the hippocampal-dependent learning and memory were evaluated using Morris water maze tests in our study. Herein, 1-week-old mice were exposed to isoflurane (1.5%) for 6 h first. 3 weeks later, the mice were used for learning and memory test. The escape latency was clearly prolonged by isoflurane exposure (Fig. 6A, B). In addition, isoflurane also decreased the time spent in the target quadrant as well as the number of plateform crossing in 60 s (Fig. 6C, D), indicating the impairment on learning and memory. The pre-treatment with either Fer-1 or DMF rescued the isoflurane-induced learning and memory impairment, as the mice in the Fer-1+ISO group or DMF + ISO group had shorter escape latency and more plateform crossing than single ISO group (Fig. 6A–D).

## Discussion

**Fig. 7:**
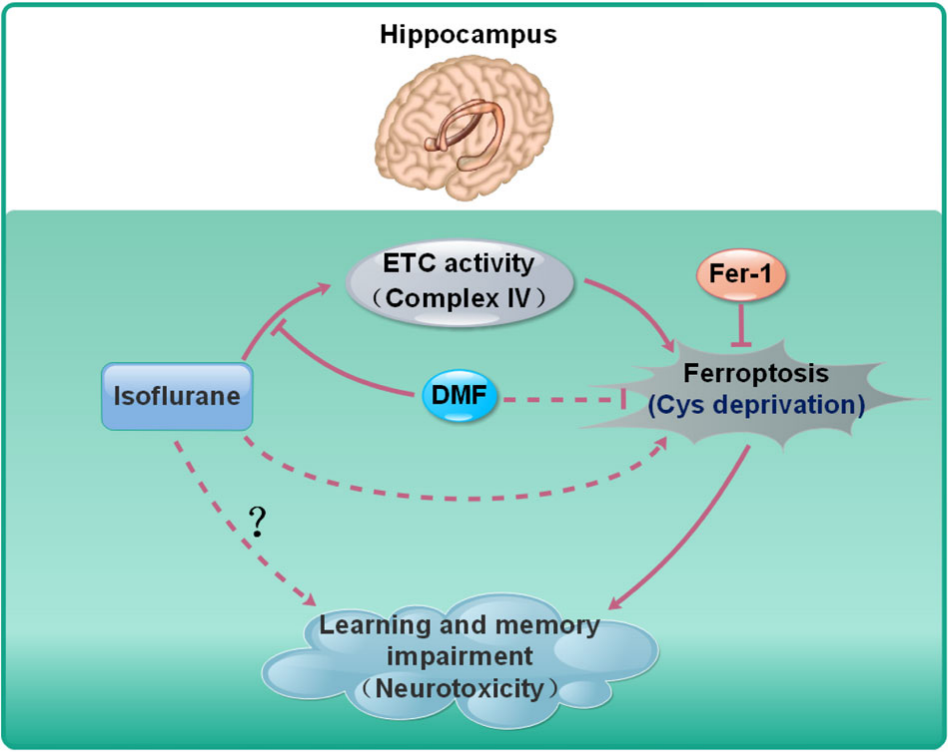
Proposed model for the relationship among isoflurane, ferroptosis and learning and memory impairment in hippocampus. Isoflurane exposure induces ferroptosis in hippocampus and leads to learning and memory impairment. Isoflurane also increased the activity of cytochrome c oxidase/Complex IV in mitochondrial ETC, which is essential for cysteine deprivation-induced ferroptosis. The effect of isoflurane on Complex IV can be rescued by DMF, but not Fer-1.

Ferroptosis is novel type of programmed cell death, and characterized by iron-dependent accumulation of lipid peroxidation. This concept was first defined by Dr. Stockwell group in 2012^17^. As early as 2010, some researchers had noticed that the volatile anesthetic, desflurane, decreased the level of vitamin E and enhanced lipid peroxidation and oxidative stress in patients’ blood^38^. Therefore, ferroptosis could be related with the side effects of volatile anesthetic. The position of ferroptosis in isoflurane-induced neurotoxicity was explored by some groups in 2019. The scientists found the cellular chelatable iron and lipid peroxidation were upregulated by isoflurane exposure, and the ferroptosis inhibitors, Fer-1 and deferoxamine mesylate, hold a strong potential to maintain the viability of neural cells exposed to isoflurane in vitro^16^. However, the in vivo significance of ferroptosis in isoflurane-induced neurotoxicity as well as learning and memory impairment is still unclear. Thus, in our study, we mainly analyzed the in vivo effect of isoflurane on ferroptosis in hippocampus that was related with learning and memory ability. We found that isoflurane exposure indeed induced ferroptosis in hippocampus via affecting system Xc−. Moreover, the activity of cytochrome c oxidase/Complex IV in mitochondrial ETC was also increased by isoflurane, and the enhanced mitochondrial ETC might further contributed to cysteine deprivation-induced ferroptosis caused by isoflurane exposure. In addition, isoflurane-induced ferroptosis as well as learning and memory impairment, could be rescued by either Fer-1 or DMF, indicating the therapeutic action of ferroptosis inhibitors and mitochondria protective agents against isoflurane-induced neurotoxicity (Fig. 7).

The effects of isoflurane on mitochondria have been investigated by different groups. Most studies indicates that isoflurane exposure can cause mitochondrial dysfunction^35,37^. However, the detailed mechanisms are still debatable. For example, Sanchez et al. found that isoflurane exposure resulted in the enlargement of mitochondria and impaired mitochondrial morphogenesis in brain. Similar to our study, their results also indicated that the activity of cytochrome c oxidase/Complex IV was increased by isoflurane, and isoflurane didn’t affect the activity of Complex I–III in mitochondrial ETC^35^. However, Hirata et al. showed different results. In their study, they found that isoflurane produced more ROS at the Complex I and Complex III in mitochondrial ETC, which depended on the attenuation of Complex I activity. Therefore, isoflurane could exposure led to the mitochondrial dysfunction via forward versus reverse electron which was regulated by Complex I, and didn’t affect the activity of Complex II–IV in mitochondrial ETC^39^. In addition, another group showed that isoflurane exposure increased the activity of Complex I–III, but decreased the the activity of Complex IV in mitochondrial ETC^40^. Those controversial conclusions may be because the animal models used in each study are different, and the dose and time for isoflurane exposure also vary a lot. Therefore, more convinced models are still essential to analyze the accurate effects of isoflurane on mitochondrial function.

Learning and memory are regarded as the important aspects of cognitive ability. Some studies have demonstrated that showed that either prenatal or postnatal exposure to isoflurane can lead to the deficits in learning and memory ability^41^. In addition, neuroapoptosis is considered as the major factor for isoflurane-induced neurotoxicity as well as learning and memory impairment. After isoflurane exposure, the levels of cleaved Caspase-3 and other apoptotic proteins were increased in hippocampal regions, while the expression of Bcl-2 (anti-apoptotic protein) was severely inhibited by isoflurane^42,43^. Therefore, the anti-apoptotic agents hold a promising potential against isoflurane-induced neurotoxicity and learning and memory impairment. In our study, we identified ferroptosis as an important contributor to isoflurane-induced neurotoxicity. Both Fer-1 (ferroptosis inhibitors) and DMF (mitochondria protective agents) showed the therapeutic action against isoflurane-induced neurotoxicity. Beside mitochondria protective function, DMF also holds effective antioxidant and anti-inflammatory function, and inhibits apoptosis because of its multiple targets^44–46^. However, it is very hard to evaluate the ratio of contribution from ferroptosis and apoptosis in isoflurane-induced neurotoxicity, since they are related to each other, and both of them can be regulated by mitochondrial pathway. Therefore, more specific and effective models are still essential for the further investigation in this field.

## Conclusion

In conclusion, our study mainly indicated the isoflurane exposure indeed induced ferroptosis in hippocampus via affecting system Xc^−^. More importantly, we found the activity of cytochrome c oxidase/Complex IV in mitochondrial ETC was increased by isoflurane, which might further contributed to cysteine deprivation-induced ferroptosis caused by isoflurane exposure. Besides, the pre-treatment with either ferroptosis inhibitor (Fer-1) or mitochondria protective agent (DMF) relived isoflurane-induced ferroptosis as well as learning and memory impairment. Even though the the crosstalk among isoflurane, ferroptosis and learning and memory impairment needs more investigation, our current research provides a novel therapeutic strategy against the neurotoxicity induced by isoflurane.

## Materials and methods

### Animal experiments

All mice were handled according to the Guide for the Care and Use of Laboratory Animals, and the protocols have been approved by the Ethics of Human Subject Research and Animal care of Jinan University (Guangzhou, China). 1-week old male C57/BL6 mice (Charles River) were randomly assigned to each group (*n* = 5). Anesthesia procedure was similar to the references^37,47^. In brief, the mice in the anesthesia group received isoflurane (0.5%, 1.0% and 1.5%, PIR001325-EA, SAS) in 100% oxygen for 2 h, 4 h and 6 h respectively in an anesthetizing chamber. In the control group, the mice only received 100% oxygen in an identical chamber. In the pre-treatment, 15 mg/kg DMF (242926, Sigma) was administered (i.p.) 16 h before anesthesia, and 1 pmol of Fer-1 (SML0583, Sigma) was injected into the striatum immediately before anesthesia. Herein, no blinding was designed.

The mice breathed spontaneously. In addition, both isoflurane and oxygen concentrations were measured continuously (Datex). In the anesthetizing chamber, the temperature was kept at 37 ± 0.5 °C. After anesthesia, all of mice were sacrificed immediately, and the whole hippocampus was isolated and homogenated for different measurements rapidly as the reference^48^.

### Real-time quantitative PCR (qRT-PCR)

In our study, Qiagen Rneasy Mini Kit (74104, Qiagen) was used to extract total RNA and the cDNA was synthesized using 2 μg of total RNA and the First Strand cDNA Synthesis Kit (K1641, Thermo Scientific). Finally the qPCR was performed using PowerUp^™^ SYBR^™^ Green Master Mix (A25742. Thermo Fisher). *Actb gene was* used as the reference for sample normalization. The primer sequences used in our study are as follows (5ʹ–3ʹ):

*Ptgs2*-F CTGCGCCTTTTCAAGGATGG

*Ptgs2*-R GGGGATACACCTCTCCACCA

*Actb*-F AAATCGTGCGTGACATCAAAGA

*Actb*-R GCCATCTCCTGCTCGAAGTC

### Detection of malondialdehyde (MDA), 4-hydroxynonenal (4-HNE) and glutathione (GSH)

The level of MDA, 4-HNE and GSH were measured to evaluate ferroptosis in different groups. Herein, the concentration of MDA, 4-HNE and GSH in tissue lysates were measured with the MDA Assay Kit (MAK085, Sigma-Aldrich), 4-HNE Assay Kit (ab238538, Abcam), and QuantiChrom^™^ GSH Assay Kit (DIGT250, Bioassay Systems) according to the manufacturer’s instructions respectively.

### Western blot analysis

The cell samples were harvested using RAPI buffer containing PMSF (11359061001, Sigma) and Protease Inhibitor Cocktails (P8849, Sigma), and were subjected to western blot assay as our previous description^49,50^. Herein, the antibody against SLC7A11 (1:1000, 12691 S) was purchased from Cell Signaling Technology, and the antibodies against GPX4 (1:1000, sc-166570) and TIM23 (1:1000, sc-514463) and GAPDH (1:3000, sc-32233) were purchased from Santa Cruz Biotechnology. Finally, the intensity of each signal was calculated using a Quantity One Image Program (Bio-Rad).

### Evaluation of mitochondrial ETC activity

In this study, mitochondria was first isolated from tissue samples with Mitochondria Isolation Kit for Tissue (ab110169, Abcam). Then, the activity of Complex I–IV in mitochondrial ETC was measured using Complex I Enzyme Activity Assay Kit (ab109721, Abcam), Complex II Enzyme Activity Assay Kit (ab109908, Abcam), Mitochondrial Complex III Activity Assay Kit (K520-100, Biovision), and Complex IV Enzyme Activity Assay Kit (ab109909, Abcam) according to the manufacturer’s instructions respectively.

### Morris water maze test

The Morris water maze test was used to evaluate the learning and memory ability in each group. 1-weel old mice received isoflurane exposure and pretreatment first. 3 weeks later, the learning and memory ability of the mice (weight = 15 ± 1 g) in different groups were analyzed. A round pool (diameter: 150 cm; depth: 50 cm) was filled with warm water (25 ± 1 °C). In addition, the escape platform (diameter: 10 cm) was fixed in the middle of one of the quadrants, and was placed at 1 cm below the water surface. The mice were trained in two sessions/day for 5 consecutive days. Throughout the training period, the mice were allowed to swim in the pool freely until they reached the platform the escape platform, which was placed in the same position (target quadrant). The time taken by the mice in each group to reach the escape platform was recorded as the escape latency. Besides, 24 h after the training phase, the mice swam freely in the water pool for 2 min, and the time spent in the target quadrant was calculated. Moreover, the number of plateform crossing in 60 s was also recorded in each group.

### Statistics

In our study, all of data were expressed as mean ± SD (*n* = 5). Statistical analysis was performed using Graphpad Prism 8.0. Unpaired Student’s *t* tests were used to compare the difference of two groups, and one-way ANOVA with Bonferroni’s correction was used to compare the means of three or more groups. *P* value < 0.05 was considered statistically significant.
